# How do improved lentil varieties affect farmers’ livelihood status in central Bangladesh?

**DOI:** 10.1016/j.heliyon.2024.e41189

**Published:** 2024-12-13

**Authors:** Monira Sultana, Jannatul Ferdous Shetu, Fatema Sarker, Sharif Ahammad, Md. Hayder Khan Sujan

**Affiliations:** a Department of Agricultural Economics, Sher-e-Bangla Agricultural University, Dhaka, Bangladesh; b Department of Development and Poverty Studies, Sher-e-Bangla Agricultural University, Dhaka, Bangladesh; c School of Agriculture and Food Sustainability, The University of Queensland, Gatton, QLD, 4343, Australia

**Keywords:** Asset pentagon, Livelihood assessment index, Profitability, Pulses, Sustainable livelihood framework, Bangladesh

## Abstract

Lentil plays a crucial role in ensuring nutritional security for the majority of the people in Bangladesh. Different types of improved lentil varieties (ILVs) are being introduced in Bangladesh to enhance the production, productivity, and area coverage. This study aimed to explore the changes in the livelihood status of the ILVs adopters compared to the non-adopters in the central region of Bangladesh. This study constructed a livelihood assessment index (LAI) by adopting the sustainable livelihood framework (SLF) provided by the Department for International Development (DFID) using primary data collected from two hundred randomly selected respondents during March–April 2022. LAI scores of the ILVs adopters and non-adopters were 0.48 and 0.43, respectively, indicating better livelihood status of the adopters. Major changes were observed in the human, natural, and physical capital of the lentil growers. ILVs adopters drew a profit margin of 662.46 USD/ha, which was 298.24 % higher compared to the non-adopters. Moreover, they realized a 47.32 % and 6.75 % rise in productivity and cost of production, respectively. Besides the positive impact on the livelihood status, ILVs adoption was remunerative from the agronomic and economic perspectives. To optimize the benefits of ILVs, strategies such as demonstrating their tangible advantages and providing agricultural input and output supports are crucial to expedite better adoption by farmers. Additionally, research is required for fitting short-duration lentil varieties in between the rice-based cropping patterns in Bangladesh.

## Highlights

• Improved lentil varieties (ILVs) adopters’ livelihood status change was assessed by the Livelihood Assessment Index (LAI).
• The ILVs adopters had better livelihood status, with LAI scores of 0.48 compared to the non-adopters (LAI, 0.43).
• The adopters drew a profit margin of 662.46 USD/ha, which was 298.24 % higher than the non-adopters.
• Studies are warranted for better-fitting short-duration lentil varieties in between the rice-based cropping patterns.

## Graphical abstract

Graphical abstract on “How do improved lentil varieties affect farmers’ livelihood status in central Bangladesh?”.

**Figure:**
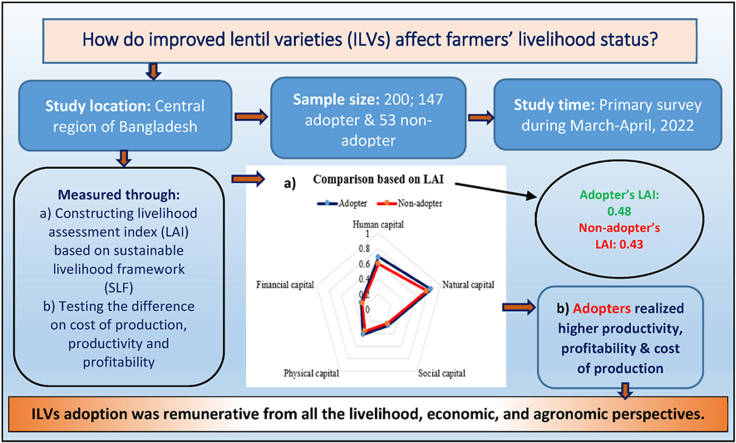

## 1 Introduction

Lentil acts as a crucial element of the human diet, supplying proteins, essential amino acids, dietary fiber, minerals, and vitamins, contributing to global food and nutritional security [1,2]. In Bangladesh, lentil is the most significant pulse crop, used for human consumption, animal feed, and as a raw material for the agro-processing industries [3]. It is the most widely planted pulse crop in Bangladesh, covering 40 % of the total pulse-producing area and 45 % of total production. It is grown in different places around the country, accounting for 146 thousand hectares of land and producing 185.50 thousand metric tons of yield with a productivity of 1.27 t/ha [4]. Winter, specifically November to March, is the best time to grow lentil. It requires minimum tillage and fewer agricultural practices, such as weeding and little to no irrigation [5]. It can be cultivated as a single, mixed, or intercrop. Between 1991 and 2015, BARI and BINA released 15 improved lentil varieties (ILVs). The emergence of the ILVs has increased the appeal of cultivating lentil in rice-based relay cropping systems because of their shorter growth duration.

Bangladesh's people consume only 17.1 g of pulse per capita per day, which is far below the World Health Organization (WHO)'s recommended portion size, which is 45 g [6]. To meet the growing demand for pulses, ILV's adoption by farmers could help address the problem. Compared to local cultivars, the improved variety generates higher yield and profitability [7,8]. The adoption of improved varieties contributes positively to dietary diversity and decreases food insecurity [9].

Among the very few studies in Bangladesh, Yigezu et al. (2022) found the positive capacity of ILVs in increasing yields and gross margins [8]. In an explorative study, Miah et al. (2022) found that about 71 % of farmers adopted ILVs in the Faridpur, Magura, Kushtia, Jhenaidah, Manikganj, and Sirajganj districts [10]. Among the adopted ILVs, BARI Masur-6 was the most widely cultivated variety, followed by BARI Masur-4, -3, and -5 [11]. In another study, Rahman et al. (2018) explored the adoption status of ILVs in Bangladesh [12]. Besides, Yigezu et al. (2019) investigated the determinants of modern lentil varieties adoption and their impact on the plot-level yield performance in Bangladesh [13]. Hasan et al. (2008) estimated the average technical efficiency of improved pulse production in Bangladesh with and without considering environmental factors and found the score was 79.8 % and 64.7 %, respectively [14]. However, in Nepal, the mean technical efficiency in lentil production was found 61.5 % [15]. Praharaj et al. (2021), Gautam et al. (2016), and Dogra et al. (2016) found that the livelihood status of the Indian pulse farmers improved with the adoption of improved varieties [[16], [17], [18]]. Similarly, in Bangladesh, Sharna et al. (2020) conducted a similar study on improved chickpea cultivators and identified their positive contribution to their livelihood development [19]. This state of art reveals that the contribution of ILVs towards the improvement of farmers' livelihoods remains unexplored. With this background, this study was undertaken to answer the research question, “How does the livelihood status of the ILV adopters differ from that of non-adopters in Bangladesh?” The specific objectives of this study were a) to explore the livelihood status of the ILVs adopter and non-adopter farmers; b) to compare their livelihood status; and c) to compare the change in profitability due to the adoption of ILVs. The findings of this study will help the policymakers craft or reshape the strategies based on the contribution of ILVs to people's livelihoods. It will also guide the researchers and extension personnel in their future studies and actions for the betterment of the farmers and lentil consumers of the country.

## 2 Methodology

### 2.1 Study site

**Fig. 1:**
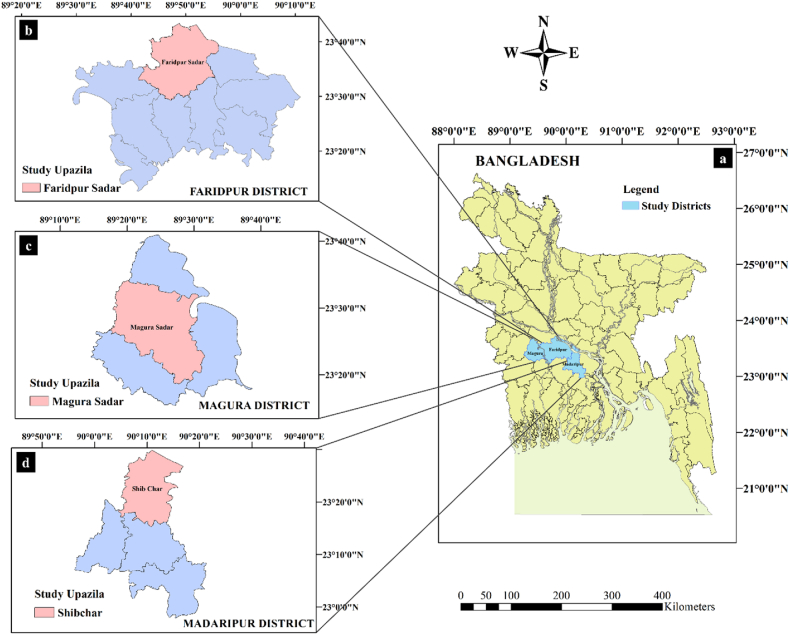
Map of the study area. a) Map of Bangladesh showing study districts Faridpur, Magura, and Madaripur. b) Map of Faridpur district showing study sub-district Faridpur Sadar. c) Map of Magura district showing study sub-district Magura Sadar. d) Map of Madaripur district showing study sub-district Shibchar. The maps were created using ArcGIS version 10.8 adopting the shape file published by the Bangladesh Agricultural Research Council (BARC).

Lentil is grown well in the different regions of Bangladesh. However, it is extensively cultivated in the mid part of the country [20]. Besides, cultivable land in this region is plain, which is the most regular land type in Bangladesh. For this reason, this study was conducted in the mid-central region purposively. From that part, Faridpur, Magura, and Madaripur districts were chosen based on their higher level of production. In the third step, the Faridpur Sadar, Magura Sadar, and Shibchar subdistricts from the Faridpur, Magura, and Madaripur districts were selected, respectively. In the final step, lentil farmers were selected randomly. Details about the study area are presented in Fig. 1.

### 2.2 Sampling and sample size

All the lentil farmers in the Madaripur, Faridpur, and Magura districts constituted the population of the study. The actual number of lentil growers is unknown. So, the sample size for this study was determined using the sample size calculation formula for the unknown population (Eq. (1)). Different values on the sample size determination formula were chosen following Kanyenji et al. (2020) and Rahman et al. (2021) [21,22].

(1) $$n=\frac{z^{2}p\left(1-p\right)}{d^{2}}=\frac{\left(1.96^{2}\right)*0.5\left(0.5\right)}{{\left(0.07\right)}^{2}}=196≅200$$

where n refers to the sample size, and z refers to the confidence level. The commonly used confidence level is 95 %, and for that the z value is 1.96. p refers to the estimated population proportion, which is taken as 50 % (p=0.5) as that helps maximize the sample size. d refers to the desired precision level, which is assumed to be ±7 %. Eq. (1) suggests that a sample of 196 respondents was enough. For better clarity, a total of 200 lentil farms were surveyed randomly to collect quantitative data during March–April 2022. From the study region, 70, 70, and 60 farms of Faridpur Sadar, Magura Sadar, and Shibchar subdistricts were surveyed. The number of farms surveyed from the Shibchar subdistrict was lower, as it produces a lower amount of lentil than the other two. Random survey data revealed that among the respondents, improved lentil variety adopters and non-adopters were 147 (73.50 %) and 53 (26.50 %), respectively. Before administering the final survey, the interview schedule was scrutinized to address the problems faced during the pre-test. The questionnaire was further developed having scholarly comments from the experts. Details on the sample are presented in Table 1.

**Table 1:** Details of the study area.

District	Upazila	Number of sample
Faridpur	Faridpur Sadar	70 (35.00)
Magura	Magura Sadar	70 (35.00)
Madaripur	Shibchar	60 (30.00)
Total		200 (100.00)

Note: Numbers in the parenthesis indicate the percentage of total sample.

### 2.3 Analytical methods

To compare the livelihood status of the ILVs adopters and non-adopters, a livelihood assessment index (LAI) was constructed adopting the sustainable livelihood framework (SLF) provided by the Department for International Development [23]. According to this framework, livelihood is a function of the following five capitals:

Livelihood=f(human,natural,social,physical,andfinancialcapital)

To assess and compare the livelihood status, a composite index was developed based on these five major livelihood components. Subcomponents of these capitals were estimated and compared by assigning similar weightage to build complete scenarios for the ILVs adopters and the non-adopters [24]. The subcomponents for this study were selected based on the knowledge that existed in open-sourced literature and field experiences (Table 2). Although five major capitals comprise a varied number of subcomponents, the accumulated score for each of them to the composite index was equal. To escape any measurement bias by using different scales for different subcomponents, standardization was done using Eq. (2):

(2) $${\text{Index}}_{\text{SA}=}\frac{\left(S_{A}\;–\;S_{\min}\right)}{\left(S_{\max}-S_{\text{mi}n}\right)}$$

where, $S_{A}$ refers to the original subcomponent value at situation A, $S_{\min}$ and $S_{\max}$ refer to the minimum and maximum value of the subcomponents, respectively.

**Table 2:** Description of the livelihood components.

Component of livelihood	Sub-components	Justification	Value	Sources
1. Human capital	Number of income-earning people in the family	A higher number of income-earning people in the family can increase income and livelihood status.	Number	[26]
	Participation in agricultural training	Participation in training can enhance knowledge and livelihood status.	Yes = 1 No = 0	[27]
	Access to timely healthcare facilities	Access to healthcare facilities on time can enhance health capital.	Yes = 1 No = 0	[28]
	Access to nutritious food	Access to nutritious food can improve health status and livelihood.	Yes = 1 No = 0	[24]
	Child schooling	Education helps to grab greater income-earning opportunities and livelihood.	Yes = 1 No = 0	[29]
2. Natural capital	Access to safe drinking water	Access to safe drinking water helps maintain a healthy life.	Yes = 1 No = 0	[30]
	Cropland ownership	Cropland ownership can ensure better income and livelihood.	Yes = 1 No = 0	[31]
	Livestock and poultry ownership	Livestock and poultry ownership is also helpful to diversity and enhance income and livelihood status.	Yes = 1 No = 0	[24]
3. Social capital	Connected with agricultural cooperatives	Connection with agricultural cooperatives can enhance income-earning opportunities and livelihood.	Yes = 1 No = 0	[24,32]
	Access to social safety net program	Access to social safety net programs may improve livelihood conditions.	Yes = 1 No = 0	[33,34]
	Communication with the extension officer	Regular extension contact can improve crop cultivation-related knowledge, income, and livelihood status.	Yes = 1 No = 0	[32]
	Societal organizational participation	Societal organizational participation can improve social capital.	Yes = 1 No = 0	[25,32]
4. Physical capital	Ownership of house	Housing status is an important indicator of livelihood status.	Yes = 1 No = 0	[31]
	Ownership of agricultural machinery	Ownership of agricultural machinery can influence income and livelihood status.	Yes = 1 No = 0	[35]
	Ownership of vehicles	Access to transportation facilities can ensure income from diversified sources.	Yes = 1 No = 0	[31]
5. Financial capital	Income from service	Income from the service sector can stabilize and enhance financial capital.	Yes = 1 No = 0	[24]
	Allows women to work outside the home	Allowing women to work outside the home can enhance income-earning opportunities and better livelihood status.	Yes = 1 No = 0	[36]
	Income from business	Income from business can enhance the opportunity of having a diversified income.	Yes = 1 No = 0	[37]
	Have savings	Savings are an important sign of improved livelihood.	Yes = 1 No = 0	[36]
	Access to formal credit	Access to credit can improve command over unexpected financial situations.	Yes = 1 No = 0	[24]

Once completed the estimation of index value for the subcomponents, the index for each livelihood component was estimated using the following Eq. (3):

(3) $$M_{\text{AJ}}=\frac{\sum_{i=1}^{n}{\text{Index}}_{\text{SAi}}}{n}$$

where $M_{\text{AJ}}$ refers to the index value of the major component J for situation A, ${\text{Index}}_{\text{SAi}}$ refers to the value of subcomponents, indexed by i, of the major component $M_{J}$; and n refers to the number of subcomponents in the major component $M_{J}$.

After calculating the index value for each of the five major capitals, the LAI for situation A was calculated using the following Eq. (4):

(4) $${\text{LAI}}_{A}=\frac{\sum_{i=1}^{n}{W_{\text{MJ}}M}_{\text{AJ}}}{\sum_{i=1}^{n}W_{\text{MJ}}}$$

Eq. (4) can be extended as the following Eq. (5):

(5) $${\text{LAI}}_{A}=\frac{W_{H}H_{A}+W_{N}N_{A}+W_{S}S_{A}{+W}_{P}P_{A}+W_{F}F_{A}}{W_{H}+W_{N}+W_{S}+W_{P}+W_{F}}$$

where ${\text{LAI}}_{A}$ is the livelihood assessment index of situation A; $W_{\text{MJ}}$ refers to the weight of component J; and $W_{H},W_{N},W_{S},W_{P,}\;\text{and}\;W_{F}$ refer to the weightage given at human, natural, social, physical, and financial capital, respectively. $H_{A}$, $N_{A}$, $S_{A}$, $P_{A,}$ and $F_{A}$ refer to the index value estimated for human, natural, social, physical, and financial capital in situation A. Details of the calculation process is presented in Appendix A.

In this study, the human capital index comprises the number of earning persons in a family $\left(H_{E}\right)$, participation in agricultural training $\left(H_{\text{AT}}\right)$, timely access to health $\left(H_{H}\right)$, access to nutritious food $\left(H_{N}\right)\text{,}$ and access to child schooling $\left(H_{S}\right)$ and was constructed following Eq. (6):

(6) $$\text{Human}\;\text{capital}\;\text{index}\;\left(H_{A}\right)=\frac{W_{E}H_{E}+W_{\text{AT}}H_{\text{AT}}+W_{H}H_{H}{+W}_{N}H_{N}{+W}_{S}H_{S}}{W_{E}{+W}_{\text{AT}}{+W}_{H}{+W}_{N}{+W}_{S}}$$

where, $W_{E}{,W}_{\text{AT}},{\;W}_{H}{,W}_{N,}{\;\text{and}\;W}_{S}$ refer to the weightage assigned to the number of earning persons in the family $\left(H_{E}\right)$, participation in agricultural training $\left(H_{\text{AT}}\right)$, timely access to health $\left(H_{H}\right)$, access to nutritious food $\left(H_{N}\right)$ and access to child schooling $\left(H_{S}\right)$ subcomponents, respectively.

The index for natural capital comprises access to safe drinking water $\left(N_{\text{Dw}}\right)$, cropland ownership $\left(N_{\text{CW}}\right)\text{,}$ and livestock and poultry ownership $\left(N_{\text{LPW}}\right)$ and was constructed following Eq. (7):

(7) $$\text{Natural}\;\text{capital}\;\text{index}\;\left(N_{A}\right)=\frac{W_{\text{DW}}N_{\text{DW}}+W_{\text{CW}}N_{\text{CW}}+W_{\text{LPW}}N_{\text{LPW}}}{W_{\text{DW}}{+W}_{\text{CW}}{+W}_{\text{LPW}}}$$

where, $W_{\text{DW}}{,W}_{\text{CW},}{\;\text{and}\;W}_{\text{LPW}}$ refer to the weightage assigned to the access to safe drinking water $\left(N_{\text{Dw}}\right)$, cropland ownership $\left(N_{\text{CW}}\right)\text{,}$ and livestock and poultry ownership $\left(N_{\text{LPW}}\right)$ subcomponents, respectively.

The social capital index comprises connectedness with agricultural cooperatives $\left(S_{\text{AC}}\right)$, access to social safety net program $\left(S_{\text{SSNP}}\right)$, visiting extension officer $\left(S_{E}\right)\text{,}$ and participation in the societal organization $\left(S_{\text{SO}}\right)$ and was constructed following Eq. (8):

(8) $$\text{Social}\;\text{capital}\;\text{index}\;\left(S_{A}\right)=\frac{W_{\text{AC}}N_{\text{AC}}+W_{\text{SSNP}}N_{\text{SSNP}}+W_{E}N_{E}+W_{\text{SO}}N_{\text{SO}}}{W_{\text{AC}}{+W}_{\text{SSNP}}{+W}_{E}+W_{\text{SO}}}$$

where, $W_{\text{AC}}{,W}_{\text{SSNP}}{,W}_{E,}\;\text{and}\;W_{\text{SO}}$ refer to the weightage assigned to the connectedness with agricultural cooperatives $\left(S_{\text{AC}}\right)$, access to social safety net program $\left(S_{\text{SSNP}}\right)$, visiting extension officer $\left(S_{E}\right)\text{,}$ and participation in societal organization $\left(S_{\text{SO}}\right)$ subcomponents, respectively.

The physical capital index comprises ownership of houses $\left(P_{H}\right)$, ownership of agricultural machinery $\left(P_{\text{AM}}\right)\text{,}$ and ownership of vehicles $\left(P_{V}\right)$ and was constructed following Eq. (9):

(9) $$\text{Physical}\;\text{capital}\;\text{index}\;\left(P_{A}\right)=\frac{W_{H}N_{H}+W_{\text{AM}}N_{\text{AM}}+W_{V}N_{V}}{W_{H}{+W}_{\text{AM}}{+W}_{V}}$$

where, $W_{H},{\;W}_{\text{AM},}{\;\text{and}\;W}_{V}$ refer to the weightage assigned to the ownership of house $\left(P_{H}\right)$, ownership of agricultural machinery $\left(P_{\text{AM}}\right)\text{,}$ and ownership of vehicles $\left(P_{V}\right)$ subcomponents, respectively.

The financial capital index comprises income from service $\left(F_{\text{IS}}\right)$, women working outside the home $\left(F_{W}\right)$, income from business $\left(F_{\text{IB}}\right)$, having savings $\left(F_{S}\right)\text{,}$ and access to formal credit $\left(F_{\text{FC}}\right)$ and was constructed following Eq. (10):

(10) $$\text{Financial}\;\text{capital}\;\text{index}\;\left(P_{A}\right)=\frac{W_{\text{IS}}N_{\text{IS}}+W_{W}N_{W}+W_{\text{IB}}N_{\text{IB}}+W_{S}N_{S}+W_{\text{FC}}N_{\text{FC}}}{W_{\text{IS}}{+W}_{W}{+W}_{\text{IB}}{+W}_{S}{+W}_{\text{FC}}}$$

where, $W_{\text{IS}}{,W}_{W}{,W}_{\text{IB}}{,W}_{S,}{\;\text{and}\;W}_{\text{FC}}$ refer to the weightage assigned to the income from service $\left(F_{\text{IS}}\right)$, women working outside the home $\left(F_{W}\right)$, income from business $\left(F_{\text{IB}}\right)$, having savings $\left(F_{S}\right)$ and access to formal credit $\left(F_{\text{FC}}\right)$ subcomponents, respectively. More details of the methodology can be found in Hahn et al. (2009) and Sarker et al. (2020) [24,25]. Besides, a paired *t*-test was done to assess the difference realized by the ILVs adopters and non-adopters in the quantitative variables such as cost of lentil production, productivity, and profit margin.

### 2.4 Ethical consideration

The ethical and institutional approval for this survey-based study was taken from the respective committee under the Sher-e-Bangla Agricultural University, Bangladesh. Respondents were formally informed about the purposes of the study, and their verbal consent on the research and publication was also taken prior to commencing the interview. The respondents were assured that “their responses will be used for research purpose only and their identity will be kept confidential”.

## 3 Results

### 3.1 Comparison on the livelihood status

The livelihood status of the ILVs adopters and non-adopters is presented in Table 3. The total livelihood index of the ILVs adopters (0.48) was higher than the non-adopters (0.43), indicating a better livelihood status for the adopters. This improvement was sourced from the higher yield and better profitability of the ILVs than the local cultivars [8].

**Table 3:** Livelihood status of the improved lentil variety adopters and non-adopters.

Sub-component of major capital	Sub-component score		Major capital	Major capital score	
	Adopter	Non-adopter		Adopter	Non-adopter
Number of earning person in the household	0.13	0.18	Human	0.69	0.60
Participation in agricultural training	0.74	0.68			
Access to timely healthcare facilities	0.91	0.64			
Access to nutritious food	0.82	0.72			
Child schooling	0.87	0.79			
Access to safe drinking water	0.94	0.85	Natural	0.85	0.78
Cropland ownership	0.87	0.70			
Livestock and poultry ownership	0.73	0.79			
Connected with agricultural cooperatives	0.09	0.04	Social	0.26	0.23
Access to social safety net program	0.06	0.23			
Communication with the extension officer	0.76	0.60			
Societal organizational participation	0.13	0.06			
Ownership of house	1.00	1.00	Physical	0.41	0.35
Ownership of agricultural machinery	0.07	0.00			
Ownership of vehicles	0.15	0.06			
Income from service	0.12	0.26	Financial	0.28	0.26
Allows women to work outside the home	0.19	0.21			
Income from business	0.13	0.13			
Have savings	0.81	0.66			
Access to formal credit	0.17	0.04			
Overall livelihood index:					
Adopters	0.48				
Non-adopters	0.43				

**Fig. 2:**
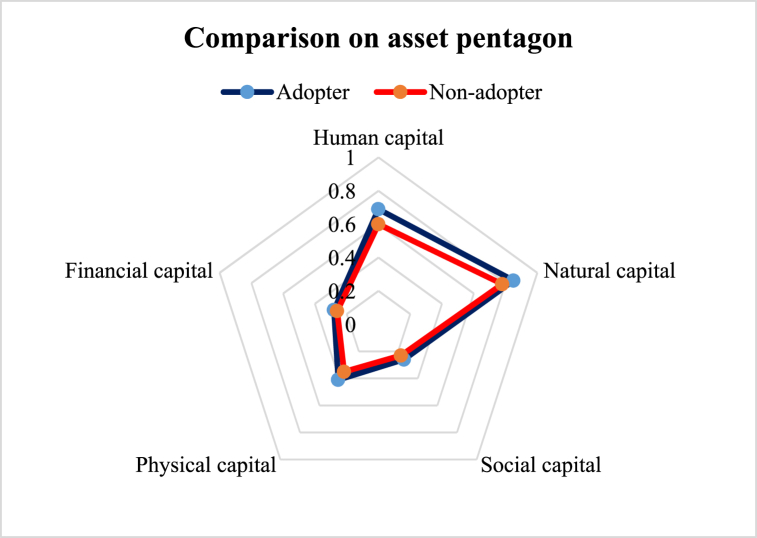
Spider diagram comparing five livelihood components of the improved lentil varieties adopter and non-adopters.

Among the five major livelihood indicators, human, natural, and physical capitals were more influenced by adopting ILVs (Fig. 2). However, the average livelihood index value for financial capital was lower because lentil production was not the main occupation of farmers. It was the added income from an additional crop. ILV's higher adaptability helped increase yield, which contributed to the livelihood improvement of the adopter farmers [38,39].

### 3.2 Comparison of the major livelihood components

#### 3.2.1 Human capital

**Fig. 3:**
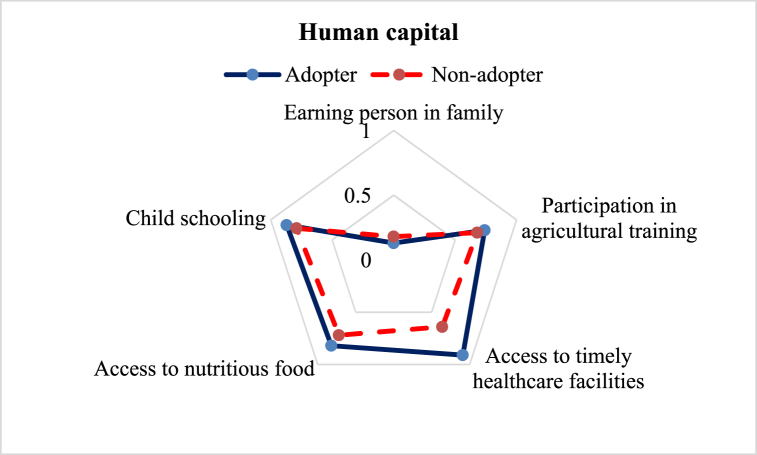
Comparison of human capital sub-component of the adopter and non-adopter of improved lentil varieties.

Human capital includes knowledge, skills, attitudes, education, mental and physical health, job capacity, and training, all of which enable individuals to achieve livelihood targets [40]. In this study, we considered five subcomponents to construct the human capital index (see Fig. 3). The estimated average human capital index of the adopters (0.69) was much greater than the non-adopters (0.60). Among the subcomponents, access to timely healthcare facilities showed the maximum variation, followed by access to nutritious food, child schooling, and participation in agricultural training. Farmer's responses revealed that about 91 %, 82 %, 87 %, and 74 % of adopters had access to timely healthcare facilities, child schooling, nutritious food, and agricultural training, respectively, but that access for the non-adopters was about 64 %, 79 %, 72 %, and 68 %, respectively (Table 3). ILV's better profitability ensures higher income for the adopters, which creates bigger opportunities for having nutritious food, better healthcare, and child schooling [41]. In case of household-earning persons, the non-adopters index (0.18) was higher than the adopters (0.13), meaning they had a greater number of income earning members in their family.

#### 3.2.2 Natural capital

**Fig. 4:**
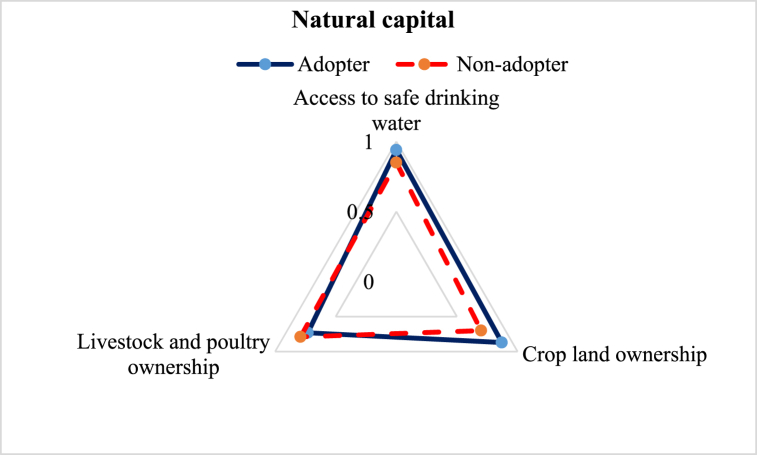
Comparison of natural capital sub-component of the adopter and non-adopter of improved lentil varieties.

Overall natural capital of the adopters was higher than the non-adopters (Fig. 4). To construct this index, we considered 3 subcomponents. The average LAI of natural capital of the adopters (0.85) was higher than the non-adopters (0.78). Results revealed that adopters (94 %) had better access to safe drinking water than non-adopters (85 %). Besides, 87 % and 73 % of the adopters owned cropland and livestock & poultry, respectively, while ownership of the non-adopters was 70 % and 79 %, respectively. The difference in the number of livestock and poultry ownership depicts that the local variety adopters were less dependent on the income from lentil cultivation to improve their livelihood status.

#### 3.2.3 Social capital

**Fig. 5:**
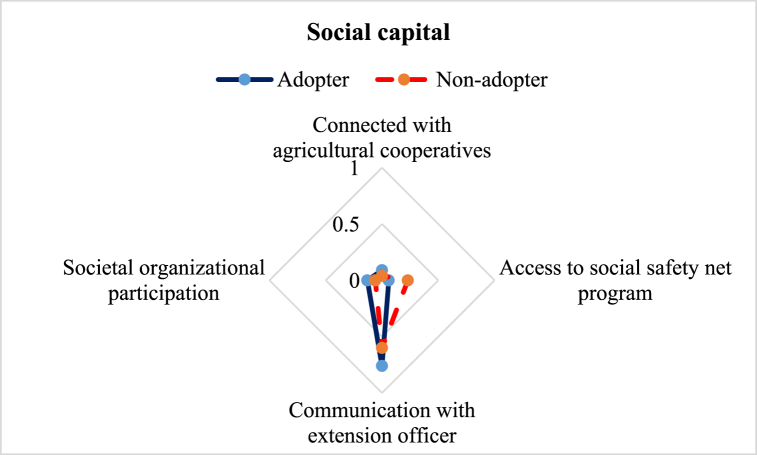
Comparison of social capital sub-component of the adopter and non-adopter of improved lentil varieties.

Difference is observed in the case of the social capital of the ILVs adopter and non-adopters (Fig. 5). In this study, we investigated 4 different subcomponents of the social capital. The average LAI of social capital for adopters (0.26) was slightly higher than for non-adopters (0.23). According to the findings, 76 % of the ILVs adopters were extension service recipients, whereas only 60 % of the local variety adopters got information from that source. Government initiatives for increasing yield through disseminating ILVs by extension workers might be the probable reason for this difference [42]. Agricultural extension officers under the Department of Agricultural Extension (DAE), Bangladesh, frequently visit the ILVs adopters to give them proper guidance for fulfilling the government objectives. The result showed that 9 % of the ILVs adopters were connected with agricultural cooperatives compared to 4 % of the non-adopters. Similarly, in the case of participation in social organizations, approximately 13 % of the adopters were connected with any organization, but that attribute was only about 6 % for the non-adopters. However, LAI on access to social safety net program (SSNP) showed different findings, where 23 % of the non-adopters had access compared to only 6 % of the adopters. Coverage under SSNP also proved the lower livelihood status of the non-adopters.

#### 3.2.4 Physical capital

**Fig. 6:**
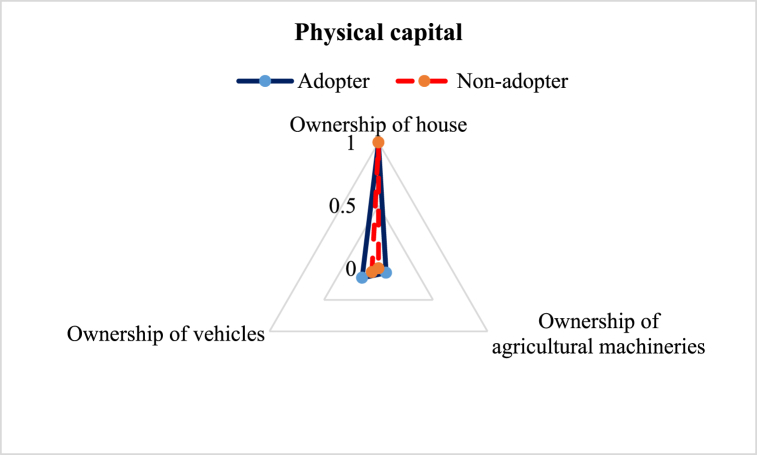
Comparison of physical capital sub-component of the adopter and non-adopter of improved lentil varieties.

Fig. 6 reveals that ILVs adopters had slightly greater average LAI on physical capital (0.41) compared to the non-adopters (0.35). We considered 3 subcomponents to construct LAI in physical capital. The findings showed no difference between the adopter and non-adopter in the case of the ownership of the house. However, only 7 % of the ILVs adopters owned agricultural machinery, which attribute was zero for the non-adopters. Farmers in Bangladesh usually do not possess the economic status of owning heavy agricultural machinery, which is the probable reason for lower LAI for both the adopters and non-adopters [19,43]. Besides, there was little difference in ownership of vehicles; adopters (15 %) owned more vehicles than non-adopters (6 %). The vehicles were mostly vans and bicycles.

#### 3.2.5 Financial capital

**Fig. 7:**
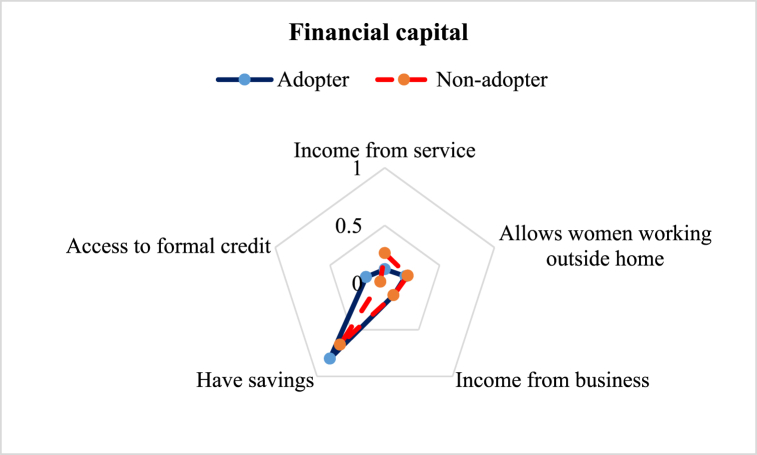
Comparison of financial capital sub-component of the adopter and non-adopter of improved lentil varieties.

The financial capital that gives people access to various livelihood alternatives is represented by their financial resources including income, savings, credits, remittances, and other assets [44]. Financial capital is a valuable asset that may be employed for the acquisition of many other forms of capital, such as natural capital (e.g., land) and physical capital (e.g., house and equipment) or human capital (e.g., education and training) [36]. In this study, we considered 5 different subcomponents to estimate the financial capital of the respondents. Results revealed that the LAI on financial capital of the ILVs adopters was (0.28) slightly higher than the non-adopters (0.26) (Table 3). Among the subcomponents of financial capital, ILVs adopters had higher savings than the non-adopters and the values were 81 % and 66 %, respectively. The adopters could save more because ILVs along with integrated crop management practices helped generate higher yields and net returns [45]. However, non-adopters had higher income from the service sector compared to the adopters, accounting for 26 % and 12 %, respectively. Service holders’ lesser priority to earn from lentil might be the reason behind their lower adoption of ILVs. As expected, adopters (17 %) had higher access to formal credit than non-adopters (4 %). Besides, about one-fifth of the adopter and non-adopters allowed their women to work outside the home. In the case of income from business, no change was observed (Fig. 7).

### 3.3 Comparative profitability of ILV adopters and non-adopters

A significant difference between the ILVs adopters and non-adopters was observed in the case of cost of production, productivity, and profitability (Table 4). Findings revealed that adopters of ILVs had a 6.75 % increment in the cost of production compared to the non-adopters. The average productivity of the ILVs adopters was 1.35 t/ha, which was 47.32 % greater than the non-adopters. Besides, the profitability of the ILVs adopters was 662.46 USD/ha, which was 298.24 % greater than the non-adopters.

**Table 4:** Comparative profitability of improved lentil variety adopter and non-adopter farmers.

Items	Non-adopter	Adopter	Mean difference	% Increased
Cost of production (USD/ha)	831.74	887.90	56.16∗∗∗	6.75
Productivity (t/ha)	0.91	1.35	0.43∗∗∗	47.32
Profit (USD/ha)	166.35	662.46	496.11∗∗∗	298.24

Note: 1 USD was equivalent to 86 BDT (Bangladeshi currency) during the production period.

## 4 Discussion

This study aimed to explore the difference in the livelihood status between the ILVs adopters and non-adopter farmers in the central part of Bangladesh. Constructed LAI reveals some variation in favor of the adopters (Table 3). Five major capitals were investigated to explore the change (Fig. 2). The assessment of human capital revealed that adopter farmers had greater access to timely healthcare facilities, child schooling, nutritious food, and agricultural training (Fig. 3), which is in line with the findings of Monwar et al. (2014) [31]. Access to agricultural training raises practical knowledge required for enhancing productivity, while timely access to food and healthcare facilities contributes to improving the farmers’ livelihood standard. Similar types of findings were also reported by Alam et al. (2016) and Ahmed et al. (2021) [28,36]. The investigation on access to natural resources revealed that adopter farmers had greater command over safe drinking water and cropland (Fig. 4). This result complies with the findings of Rahman et al. (2021) on shrimp farming in the southern coast of Bangladesh [22]. The greater natural resource gives additional income-generating opportunities that help promote sustainable livelihoods [46]. Constructed LAI on social capital depicted that ILVs adopters had greater social capital (0.26) than the non-adopters (0.23) (Fig. 5). The lower index value indicates that both groups of farmers had very limited access to different subcomponents of social capital, like access to agricultural cooperatives, SSNP, extension services, and societal organizations. This result agrees with the findings on the farming community of shrimp by Rahman et al. (2021) and fishes by Kleih et al. (2003) [22,47]. Estimated LAI on physical capital was also higher for the ILVs adopter (0.41) compared to the non-adopters (0.35) (Fig. 6), and that finding is in line with that of Ahmed et al. (2021) on the fishermen community of Bangladesh [36]. Lower LAI scores on different subcomponents of physical capital, like ownership of vehicles and agricultural machinery, reflect that there was a lack of incentive to own these physical capitals. These findings comply with those of Rahman et al. (2021) and Tikadar et al. (2022) on fishermen communities [22,48]. LAI of financial capital also showed that ILVs adopters had higher command over financial capital (0.28) compared to the non-adopters (0.26) (Fig. 7). This result is consistent with the findings of Ahmed et al. (2021), Rahman et al. (2021), and Tikadar et al. (2022) on the fishermen community [22,36,48]. However, the overall LAI was 0.48 for the ILVs adopters and 0.43 for the non-adopters. In Bangladesh, rice is the most predominant field crop, whereas people produce lentil as a relay crop, intercrop, mixed crop, etc., to enhance their agricultural income [4]. This lower contribution to the overall income, food, and livelihood might be a probable reason for the estimated lower score of LAI. The ILVs adopters fetched 1.35 t/ha, which is 47.32 % higher than the non-adopters (Table 4). However, compared to this study, Miah et al. (2021) and Yigezu et al. (2022) found higher productivity of the ILVs adopters, where they estimated the average yield as 1.58 t/ha and 1.66 t/ha, respectively [7,8]. The profit margin and the cost of ILV production were 662.46 USD/ha and 887.90 USD/ha, respectively. These results are in line with the findings of Miah et al. (2021), who estimated the profitability and the ILVs production cost as 560 USD/ha and 791 USD/ha, respectively [7]. The same things for the local varieties were also consistent with Miah et al. (2021) [7]. However, Rahman et al. (2012) found the profitability and the cost of BARI-released ILVs to be 324 USD/ha and 613 USD/ha, respectively [20]. In their study, Singh et al. (2018) showed that improved production technology enhanced the yield and net return, which helped increase the savings as well as the livelihood standard [49].

## 5 Conclusion and recommendations and future research directions

This study endeavored to explore the changes in the livelihood due to the adoption of improved lentil varieties (ILVs) in the central region of Bangladesh. The livelihood assessment index (LAI) constructed on 200 respondents revealed that ILVs adopters' livelihood status was better than the non-adopters. The estimated livelihood index also depicted the improvements in all five types of capital of the ILVs adopters. Besides, ILVs adoption helps increase the lentil production and the profit margin. However, the cost of lentil production was also increased for adopting improved varieties. Thus, the adoption of improved lentil varieties was found to be remunerative based on agronomic, economic, and livelihood aspects. To grasp the revealed benefits of ILVs, agricultural extension and community development workers should encourage local farmers to adopt the ILVs through demonstrating their tangible benefits and ensuring comprehensive input and output supports.

Assessing livelihood change based on a single crop was a limitation of this study. Thus, year-round cropping pattern-based study may help generate better inference. Besides, studies are warranted for fitting short-duration lentil varieties in between the predominant rice-based cropping patterns. Studies are also recommended for enhancing lentil production areas in Bangladesh.

## CRediT authorship contribution statement

**Monira Sultana:** Writing – review & editing, Writing – original draft, Visualization, Validation, Resources, Project administration, Methodology, Investigation, Formal analysis, Data curation, Conceptualization. **Jannatul Ferdous Shetu:** Writing – original draft, Validation, Resources, Project administration, Methodology, Investigation, Data curation, Conceptualization. **Fatema Sarker:** Writing – review & editing, Validation, Supervision, Methodology. **Sharif Ahammad:** Writing – original draft, Visualization, Data curation. **Md. Hayder Khan Sujan:** Writing – review & editing, Writing – original draft, Visualization, Validation, Supervision, Methodology, Formal analysis, Conceptualization.

## Data availability statement

Data will be made available on request.

## Additional information

No additional information is available for this paper.

Step I (repeat for all other sub-components): ${\text{Index}}_{\text{earning}\;\text{persons}\;\text{in}\;\text{family}=}\frac{1.26-1}{3-1}=0.13$

Step II (repeat for all of the major components): $\text{Human}\;\text{capital}=\frac{0.13+0.74+0.91+0.82+0.0.87}{5}=$ 0.69

Step III (repeat for both groups): ${\text{LAI}}_{A}=\frac{\sum_{i=1}^{n}{W_{\text{MJ}}M}_{\text{AJ}}}{\sum_{i=1}^{n}W_{\text{MJ}}}=\frac{5*0.69+3*0.85+4*0.26+3*0.41+5*0.28}{5+3+4+3+5}=0.48$

## Funding statement

**Monira Sultana** gratefully acknowledges the **National Science and Technology Fellowship** **2020-21** (Serial number: 285) awarded by the **Ministry of Science and Technology, Bangladesh**, for supporting her Master of Science research.

## Declaration of competing interest

The authors declare that they have no known competing financial interests or personal relationships that could have appeared to influence the work reported in this paper.

## Figures and Tables

**Table:** Calculation of livelihood index for the ILVs adopters:

Sub-components for human capital	Sub-component values	Maximum value of the sub-component	Minimum value of the sub-component	Index value	Human capital component value
The earning person in the family	1.26	3	1	0.13	0.69
Participation in agricultural training	0.74	1	0	0.74	
Access to timely healthcare facilities	0.91	1	0	0.91	
Access to nutritious food	0.82	1	0	0.82	
Child schooling	0.87	1	0	0.87	
